# Notification that new names of prokaryotes, new combinations, and new taxonomic opinions have appeared in volume 74, part 1 of the IJSEM

**DOI:** 10.1099/ijsem.0.006271

**Published:** 2024-05-02

**Authors:** Aharon Oren, Markus Göker

**Affiliations:** 1 The Institute of Life Sciences, The Hebrew University of Jerusalem, The Edmond J. Safra Campus, 9190401 Jerusalem, Israel; 2 Leibniz Institute DSMZ – German Collection of Microorganisms and Cell Cultures, Inhoffenstrasse 7B, 38124 Braunschweig, Germany

This listing of names of prokaryotes published in a previous issue of the *International Journal of Systematic and Evolutionary Microbiology* (IJSEM) is provided as a service to microbiology to assist in the recognition of new names and new combinations. This procedure was proposed by the Judicial Commission [1]. The names given herein are listed according to the Rules of priority (i.e., time and date of publication of the articles on the journal’s platform and the order of valid publication of names in the original articles) [2].

**Table:** 

Order of article publication	Name/authors	Proposed as	Article no.	Page no.
1	*Candidatus* Haliotispira Sharma *et al*. 2024*	gen. nov.	006198	7
1	*Candidatus* Haliotispira prima Sharma *et al*. 2024*	sp. nov.	006198	7
2	*Shewanella goraebulensis* Lee *et al*. 2024	sp. nov.	006214	7
3	*Commensalibacter melissae* Botero and Vandamme 2024	sp. nov.	006224	1
3	*Commensalibacter communis* Botero and Vandamme 2024	sp. nov.	006224	2
3	*Commensalibacter papalotli* Botero and Vandamme 2024	sp. nov.	006224	2
4	*Actinoplanes pyxinae* Somphong *et al*. 2024	sp. nov.	006215	7
5	*Actinoplanes sandaracinus* Wang *et al*. 2024	sp. nov.	006216	7
6	*Acidovorax benzenivorans* Bedics *et al*. 2024	sp. nov.	006219	7
7	*Microbacterium memoriense* Santos *et al*. 2024	sp. nov.	006230	10
8	*Devosia rhodophyticola* Kim *et al*. 2024	sp. nov.	006223	7
8	*Devosia algicola* Kim *et al*. 2024	sp. nov.	006223	9
9	*Actinoplanes oblitus* Yushchuk *et al*. 2024	sp. nov.	006225	9
10	*Comamonas resistens* Yin *et al*. 2024	sp. nov.	006222	12
10	*Pseudomonas triclosanedens* Yin *et al*. 2024	sp. nov.	006222	12
11	*Comamonas endophytica* Yue *et al*. 2024	sp. nov.	006217	9
12	*Halospeciosus* Li *et al*. 2024	gen. nov.	006220	6
12	*Halospeciosus flavus* Li *et al*. 2024	sp. nov.	006220	7
12	*Haladaptatus caseinilyticus* Li *et al*. 2024	sp. nov.	006220	7
13	*Halomicrococcus gelatinilyticus* Hu *et al*. 2024	sp. nov.	006231	5
13	*Halosimplex aquaticum* Hu *et al*. 2024	sp. nov.	006231	6
14	*Mycolicibacterium palauense* (Nouioui *et al*. 2019) Zhu *et al*. 2024	comb. nov. [basonym: *Mycobacterium palauense* Nouioui *et al*. 2019]	006221	6
14	*Mycolicibacterium grossiae* (Paniz-Mondolfi *et al*. 2017) Zhu *et al*. 2024	comb. nov. [basonym: *Mycobacterium grossiae* Paniz-Mondolfi *et al*. 2017]	006221	7
14	*Mycolicibacterium arseniciresistens* Zhu *et al*. 2024	sp. nov.	006221	7
15	*Tardiphaga alba* Bao *et al*. 2024	sp. nov.	006238	9
16	*Mesonia profundi* Wang *et al*. 2024	sp. nov.	006211	5
17	*Rhodococcus indonesiensis* Kusuma *et al*. 2024	sp. nov.	006236	8
18	*Azospirillum isscasi* Wang *et al*. 2024	sp. nov.	006218	7
19	*Cellulomonas alba* Park *et al*. 2024	sp. nov.	006235	6
19	*Cellulomonas edaphi* Park *et al*. 2024	sp. nov.	006235	7
20	*Tepidibacter aestuarii* Pradel *et al*. 2024	sp. nov.	006237	11
21	*Deinococcus rhizophilus* Gao *et al*. 2024	sp. nov.	006232	6
22	*Limosilactobacillus galli* Rios-Galicia *et al*. 2024	sp. nov.	006210	7
22	*Limosilactobacillus avium* Rios-Galicia *et al*. 2024	sp. nov.	006210	9
22	*Limosilactobacillus pulli* Rios-Galicia *et al*. 2024	sp. nov.	006210	9
22	*Limosilactobacillus viscerum* Rios-Galicia *et al*. 2024	sp. nov.	006210	12
22	*Limosilactobacillus difficilis* Rios-Galicia *et al*. 2024	sp. nov.	006210	12
22	*Ligilactobacillus hohenheimensis* Rios-Galicia *et al*. 2024	sp. nov.	006210	12
22	*Clostridium butanoliproducens* Rios-Galicia *et al*. 2024	sp. nov.	006210	13
22	*Faecalispora* Rios-Galicia *et al*. 2024†	gen. nov.	006210	13
22	*Faecalispora anaeroviscerum* Rios-Galicia *et al*. 2024	sp. nov.	006210	13
22	*Faecalispora jeddahensis* (Lagier *et al*. 2016) Rios-Galicia *et al*. 2024	comb. nov. [basonym: *Clostridium jeddahense* Lagier *et al*. 2016]	006210	14
22	*Faecalispora sporosphaeroides* Rios-Galicia *et al*. 2024	sp. nov.	006210	14
23	*Marinimicrococcus* Yang *et al*. 2024	gen. nov.	006241	4
23	*Marinimicrococcus flavescens* Yang *et al*. 2024	sp. nov.	006241	6
24	*Enterococcus montenegrensis* Daza-Prieto *et al*. 2024	sp. nov.	006206	11
25	*Aquimarina aquimarini* Couceiro *et al*. 2024	sp. nov.	006228	9
25	*Aquimarina spinulae* Couceiro *et al*. 2024	sp. nov.	006228	9
26	*Microbulbifer bruguierae* Long *et al*. 2024	sp. nov.	006209	7
27	*Bacteria* Woese *et al*. 2024	dom. nov.	006242	2
27	*Archaea* Woese *et al*. 2024	dom. nov.	006242	2
27	*Bacillati* (Gibbons and Murray 1978) Oren and Göker 2024	regn. nov.	006242	3
27	*Fusobacteriati* Battistuzzi and Hedges 2024	regn. nov.	006242	3
2	*Pseudomonadati* (Gibbons and Murray 1978) Oren and Göker 2024	regn. nov.	006242	3
27	*Thermotogati* Battistuzzi and Hedges 2024	regn. nov.	006242	4
27	*Methanobacteriati* (Garrity and Holt 2023) Oren and Göker 2024	regn. nov.	006242	5
27	*Nanobdellati* Rinke *et al*. 2024	regn. nov.	006242	5
27	*Thermoproteati* Guy and Ettema 2024	regn. nov.	006242	5
28	*Methylocystis suflitae* Garner *et al*. 2024	sp. nov.	006239	7
29	*Bdellovibrio svalbardensis* Choi *et al*. 2024	sp. nov.	006248	9
30	*Caproicibacterium argilliputei* Zeng *et al*. 2024	sp. nov.	006246	7
31	*Halomonas citrativorans* Kothe *et al*. 2024	sp. nov.	006234	7
31	*Halomonas casei* Kothe *et al*. 2024	sp. nov.	006234	8
31	*Halomonas colorata* Kothe *et al*. 2024	sp. nov.	006234	9
32	*Citrobacter enshiensis* Zhang *et al*. 2024	sp. nov.	006154	5
33	*Luteimonas endophytica* Wei *et al*. 2024	sp. nov.	006257	6
33	*Luteimonas rhizosphaericola* Wei *et al*. 2024	sp. nov.	006257	8
33	*Luteimonas kalidii* Wei *et al*. 2024	sp. nov.	006257	8
34	*Parachitinimonas* Wang *et al*. 2024	gen. nov.	006249	6
34	*Parachitinimonas caeni* Wang *et al*. 2024	sp. nov.	006249	6
35	*Fontisubflavum* Xu *et al*. 2024	gen. nov.	006256	6
35	*Fontisubflavum oceani* Xu *et al*. 2024	sp. nov.	006256	6
36	*Massilia litorea* Lee *et al*. 2024	sp. nov.	006255	10
36	*Marinobacter salinisoli* Lee *et al*. 2024	sp. nov.	006255	10
36	*Rhodobacter xanthinilyticus* Lee *et al*. 2024	sp. nov.	006255	10
37	*Corynebacterium mendelii* Koublová *et al*. 2024	sp. nov.	006244	7

*Candidatus names are not validly published.

†The protologue does not define the type species of the genus. *Faecalisporum anaeroviscerum* was identified as the type in Fig. 4.

The following taxonomic opinions (i.e., the emendation of circumscriptions or the creation of synonyms) do not affect the status of a name under the International Code of Nomenclature of Prokaryotes. These taxonomic opinions can neither be considered as approved nor as disapproved by the International Committee on Systematics of Prokaryotes.

**Table:** 

Name/authors:	Article no.	Page no.
*Rhodococcus electrodiphilus* Ramaprasad *et al*. 2018 pro synon. *Rhodococcus ruber* (Kruse 1896) Goodfellow and Alderson 1977 (Approved Lists 1980)	006236	8
*Rhodococcus ruber* (Kruse 1896) Goodfellow and Alderson 1977 (Approved Lists 1980) emend. Kusuma *et al*. 2024	006236	9
